# Tissue derivatization for visualizing lactate and pyruvate in mouse testis tissues using matrix-assisted laser desorption/ionization-mass spectrometry imaging

**DOI:** 10.1007/s00216-024-05559-4

**Published:** 2024-10-08

**Authors:** Erika Nagano, Kazuki Odake, Shuichi Shimma

**Affiliations:** 1 Miruion inc, 7-7-20Asagi, Saito, Suita, Osaka, 5670085 Japan; 2 Department of Biotechnology, Graduate School of Engineering, Osaka University, 2-1 Yamadaoka, Suita, Osaka, 5650871 Japan; 3 Institute for Open and Transdisciplinary Research Initiatives, Osaka University, Suita, Osaka, Japan

**Keywords:** Mass spectrometry imaging, Pyruvate, Lactate, On-tissue chemical derivatization, 3-Nitrophenylhydrazine

## Abstract

Pyruvate and lactate are the final metabolites of the glycolytic system that are formed under oxygen-rich and anaerobic conditions, respectively. They play an important role in energy metabolism. Obtaining a tissue distribution image of pyruvate and lactate holds great significance in molecular biology because the glycolytic system plays an essential role in diseases, such as tumors and diabetes; microbial activities, such as alcohol production and lactic acid fermentation; and maintaining homeostasis in the gut environment. However, it is difficult to obtain images of the distribution of in vivo metabolites because of the low detection sensitivities of current methods. In this study, a novel derivatization method for pyruvate and lactate was developed using matrix-assisted laser desorption/ionization-mass spectrometry imaging (MALDI-MSI) to detect pyruvate and lactate in vivo and obtain biodistribution images. We investigated derivatization methods using readily available 3-nitrophenylhydrazine (3NPH), the addition of which improves the sensitivity of pyruvate detection, and the distribution of pyruvate in mouse testes was successfully visualized. Furthermore, the distribution of lactate in the mouse testes could be visualized, and improved detection sensitivity for the main metabolites of the tricarboxylic acid cycle was demonstrated. This derivatization method can be used to detect carboxyl-containing metabolites, including pyruvate, via MALDI-MSI. Furthermore, 3NPH forms amide bonds with carbonyl, phosphate, and carboxyl groups, suggesting the possibility of visualizing its distribution in many metabolites.

**Graphical Abstract:**

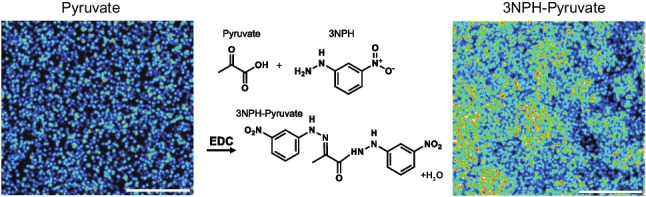

**Supplementary Information:**

The online version contains supplementary material available at 10.1007/s00216-024-05559-4.

## Introduction

Pyruvate and lactate are the final metabolites of the glycolytic system, formed under oxygen-rich and anaerobic conditions, respectively. They play an important role in energy metabolism. Pyruvate plays an important role in energy metabolism by linking the glycolytic system and tricarboxylic acid (TCA) cycle [1]. The glycolytic system is an energy metabolic pathway that occurs in nearly all living organisms and large metabolic pathways are built around it. Pyruvate is required for the biosynthesis of carbohydrates [2], lipids [3], and amino acids [4] and in fermentation processes [5–8], such as alcohol and lactic acid production. It is also considered a disease marker [9] and therapeutic target for tumors [10–12] and diabetes [9, 13] as it acts as a regulator of tumorigenesis [14, 15] and hormone secretion [16–18], such as insulin. Meanwhile, lactate is considered a waste product of metabolism under anaerobic conditions [19]. However, numerous studies have reported that it is not only essential for energy metabolism [20, 21] but also plays an important role as a signaling molecule [22–29]. As discussed above, the role of pyruvate and lactate outside of energy metabolism has attracted much attention and is often studied in diseases such as tumors and diabetes due to their anti-apoptotic effects and role in regulating sugar metabolism. However, pyruvate and lactate are also closely linked to reproductive functions. In particular, as glycolysis is the main ATP supply pathway in the mouse testis, this method was used to visualize pyruvate and lactate in the mouse testis. It showed that pyruvate and lactate in the mouse testis are distributed in the vas deferens. Since pyruvate and lactate are biosynthesized in Sertoli cells in the seminiferous tubules and are used for spermatogenesis and motility acquisition, as previously reported, the distribution image obtained by this method is considered correct, indicating that the derivatization method on tissue sections using 3NPH can be used in MALDI-MSI. In the future, metabolites will be measured and compared in infertile, and disease model mice and healthy mice to elucidate the molecular mechanisms of sperm stem cell regulation and spermatogenesis, which will be useful for developing disease markers and infertility treatments.

Mass spectrometry imaging (MSI) using matrix-assisted laser desorption/ionization (MALDI) was first reported in 1997 as a new molecular visualization method using mass spectrometry [30]. While PET and fluorescence imaging use a probe to detect the target molecule using light or electromagnetic waves, MALDI-MSI detects the target molecule itself, without a probe, using a reagent called matrix, which ionizes the molecule. This allows several molecules to be detected simultaneously in a short period of time. In particular, low molecular weight metabolites such as lipids, amino acids, and nucleic acids are often difficult to probe and only MALDI-MSI can image such metabolites simultaneously. However, in vivo, metabolites often have low detection sensitivities owing to their poor ionization efficiency and low content, and a higher sensitivity is often required to obtain distribution images. Numerous derivatization methods have been reported, including the highly sensitive derivatization of phospholipids by matrix-enhanced [ME]-Pt-SALDI, which combines inorganic and organic matrices [31–35]; methods for steroid hormones using Girard’s reagent T [36, 37]; free fatty acids using 2-picolylamine [38]; and methods for monoamines and amino acids [39, 40].

Pyruvate contains carboxyl groups, and the low biological content of its metabolites (carboxylic acids) makes it difficult to obtain distribution images. Carboxyl derivatization reagents, such as N,N,N-trimethyl-2-(piperazine-1-yl)ethan-1-aminium iodide [41] and (1-(4-(aminomethyl)phenyl) pyridine-1-mum chloride) [42], are sensitive to carboxylic acids. As these reagents require synthesis or are expensive, we investigated new derivatization methods for carboxyl-containing metabolites, including pyruvate, through MALDI-MSI using readily available reagents. In this study, 3-nitrophenylhydrazine (3NPH), which is used as a derivatization reagent for short-chain fatty acids in LC-MS/MS [43–46] was used to develop a highly sensitive derivatization method to form stable amide bonds with the carboxyl groups. Conventional derivatization methods have targeted specific functional groups per derivatization reagent, but 3NPH targets three functional groups (carboxyl, carbonyl, and phosphate groups) and is thought to allow comprehensive visualization of metabolites in the glycolytic system, the TCA cycle and the pentose phosphate pathway, the main energy metabolism. These metabolites are expected to be depicted extensively. The use of 3NPH is particularly promising as few derivatization reagents for phosphate groups have been reported [47]. In addition, when measuring compounds with phosphate groups in MALDI-MSI, a phenomenon known as in-source decay (ISD) is observed, in which the phosphate groups are removed, making it difficult to measure compounds such as ATP and ADP. We have found that ISD is suppressed by the addition of phos-tag, a known phosphate derivatizing reagent, and a similar effect can be expected with 3NPH. In addition, 3NPH can be used in both positive and negative ionic modes and could be used to detect carbohydrates and nucleic acids in negative mode and amino acids in positive mode [47].

This derivatization method was successfully used to increase the sensitivity of MALDI-MSI to pyruvate and visualize the distribution of pyruvate in mouse testes. The distribution of lactate in mouse testes was also visualized. Furthermore, an increased sensitivity to the main metabolites of the TCA cycle was confirmed. As 3NPH formed amide bonds with carbonyl and phosphate groups in addition to carboxyl groups, the possibility of visualizing its distribution in many metabolites was suggested.

## Material and methods

### Chemicals and reagents

Frozen mouse (C57Bl/6) testis tissue blocks were obtained from Kobe University. Sodium pyruvate, L-lactic acid, 1,5-diaminonaphthalen (1,5-DAN), 9-aminoacridine (9AA), 4-nitroaniline (4NA), and 3NPH were purchased from Merck (Darmstadt, Germany). 1-Ethyl-3-(3-dimethylaminopropyl) carbodiimide hydrochloride (EDC) was purchased from Tokyo Chemical Industry Co. Ltd (Tokyo, Japan). All solvents used in this experiment were of LC-MS grade and purchased from Fujifilm Wako Pure Chemical Industries Ltd.

### Tissue preparation

An optimal cutting temperature (OCT) compound was used to fix each tissue block onto a microtome holder (Leica CM 1950; Leica Microsystems, GmbH, Nussloch, Germany). The frozen tissue block was placed on the surface of the OCT compound and stored at −80 °C for 5 min. Frozen 8-μm sections were sliced at −20 °C with the microtome. The frozen sections were mounted on an indium-tin-oxide (ITO)-coated glass slide (SI0100N, Matsunami Glass, Osaka, Japan) and allowed to dehydrate in a 50-mL conical tube containing silica gel. The glass slides were stored at −20 °C in the tube until matrix application.

### Optimization of derivatization conditions for MALDI-MSI

The derivatization method was studied using previously published LC-MS methods [43, 44]. The derivatization conditions of MALDI-MSI were optimized in the liquid phase and at the tissue surface. Pyruvate was used as a standard to optimize the derivatization conditions. The pyruvate was dissolved in distilled water to a final concentration of 1 mM. 3NPH and EDC were dissolved in methanol:distilled water mixtures with ratios of 3:1 and 1:1 (v/v), respectively. The 1,5-DAN matrix was dissolved in acetonitrile and distilled water (7:3, v/v) to a final concentration of 10 mg/mL. The derivatization reaction was optimized for a single tube. The 3NPH:EDC standard was mixed at a ratio of 1:2:1 (v/v) and stirred well for derivatization. At the end of the reaction, a volume of the 1,5-DAN matrix solution that was four times larger than that of the derivatization reaction solution was added. During the derivatization reaction, the amount of the derivatization reagent supplied and the reaction environment were determined using the airbrush spray method. In the spray method study, after dropping 0.5 µL of the standard solution (62.5 µM) onto an ITO glass, the 3NPH:EDC mixture was sprayed, and the derivatization reaction was performed for 1 h under each environment. After the reaction, 100 µL of the 1,5-DAN matrix was applied. MALDI-MSI was performed after 5 min at room temperature (approximately 25 °C).

### Derivatization reactions on sections of the tissue

3NPH (200 mM) and EDC (25 mM) were dissolved in methanol:distilled water mixtures with ratios of 3:1 and 1:1 (v/v), respectively. The derivatization solution used was a 2:1 (v/v) mixture of the 3NPH and EDC solutions. For the MALDI-MSI analysis of pyruvate in the mouse testis, coronal sections were prepared with a section thickness of 8 µm, and an air brush (HT-381 0.3 mm, Wave, Tokyo, Japan) was used to spray approximately 150 µL (six cycles of 5 s/cycle) of the derivatization reagent solution evenly onto one tissue section. After spraying, the derivatization reaction was allowed to stand in gaseous acetonitrile at room temperature for 2 h to complete the derivatization reaction on the tissue.

### MALDI matrix supply

A vacuum evaporation system (iMLayer™; Shimadzu Corporation, Kyoto, Japan) was used to deposit 1,5-DAN on each ITO glass loaded with testicular tissue sections at 200 °C for 3 min. The samples were immediately measured after sublimation.

### MALDI-MSI analysis

MALDI-MSI experiment was performed on a MALDI ion trap time-of-flight mass spectrometer (iMScope™ TRIO; Shimadzu, Kyoto, Japan) [48]. The laser diameter was 25 μm. Data were collected at 10-µm intervals. The tissue surface was laser-irradiated at 80 shots (repetition rate of 1 kHz) for each pixel. All data were acquired using an external calibration method in the negative-ion detection mode. The data acquisition parameters of the laser power and collision energy were adjusted to 55 and 50 (dimensionless collision energy in iMScope™ TRIO), respectively, to obtain the maximum intensity at each target peak. The voltage of the microchannel plate detector was 2.1 kV. After sample analysis, ion images were reconstructed based on peak intensities extracted from *m/z* ranges of the target *m/z* ± 0.02 Da using IMAGEREVEAL™ MS (Shimadzu, Kyoto, Japan).

## Results and discussion

### High sensitivity to pyruvate

**Fig. 1:**
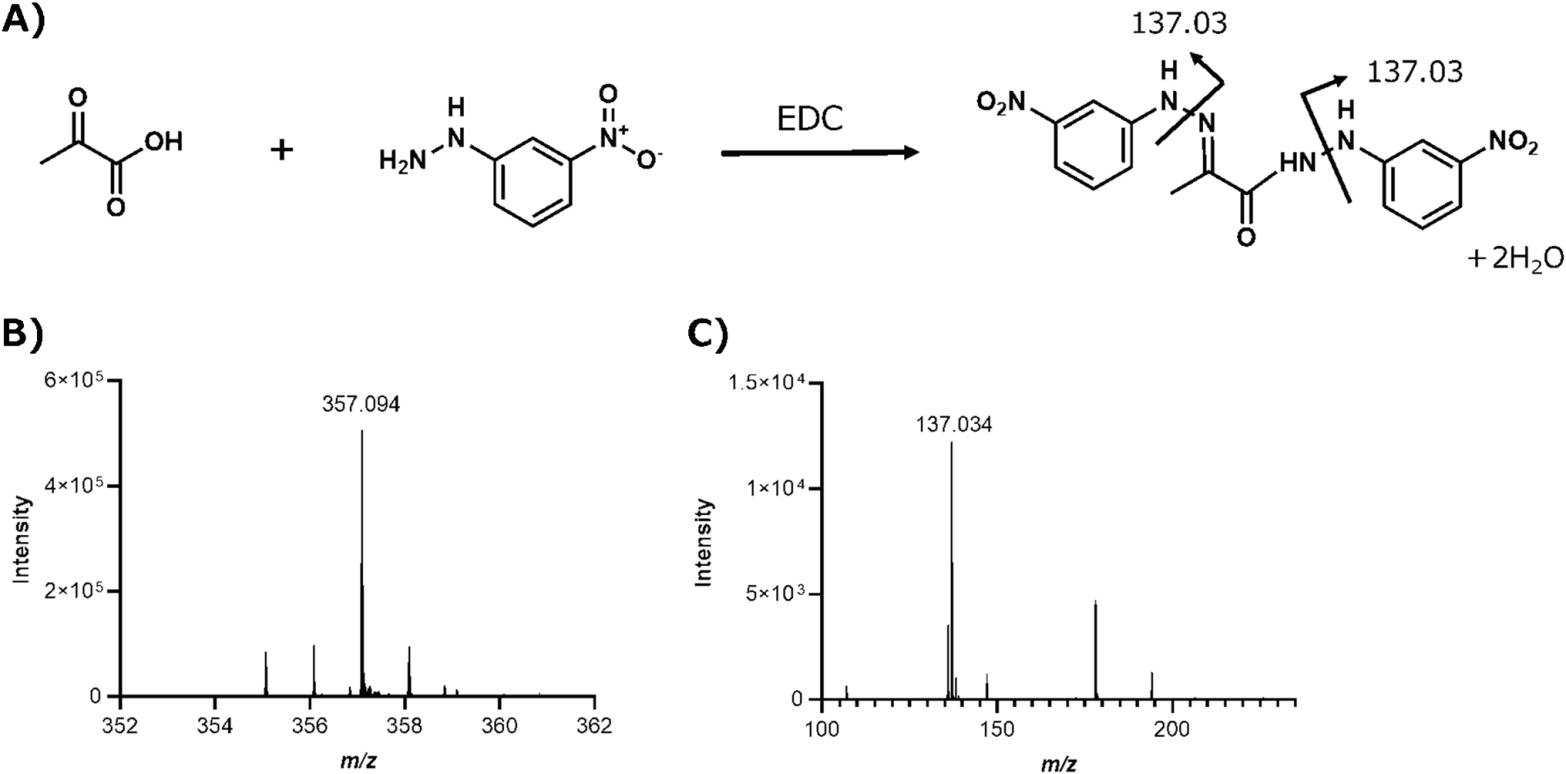
Structure and mass spectrum of 3-nitrophenylhydrazine (3NPH)-pyruvate. **A** Molecular structure of 3NPH-pyruvate formed by the reaction of 3NPH with pyruvate. **B** *m/z* 357.09 is the MS spectrum of 3NPH-PA. **C** *m/z* 137.09 is the product ion spectrum of 3NPH-PA. The carbonyl and carboxyl groups of pyruvate and the amino group of 3NPH form amide bonds by 1-ethyl-3-(3-dimethylaminopropyl) carbodiimide hydrochloride (EDC)-induced addition/elimination reactions

The structure of 3NPH-added pyruvate (3NPH-PA) is shown in Fig. 1A. In the derivatization reaction, the amino groups of 3NPH and carbonyl and carboxyl groups of pyruvate formed amide bonds. The representative mass (Fig. 1B) and product ion spectra (Fig. 1C) of 3NPH-PA were obtained via derivatization with pyruvate.

**Fig. 2:**
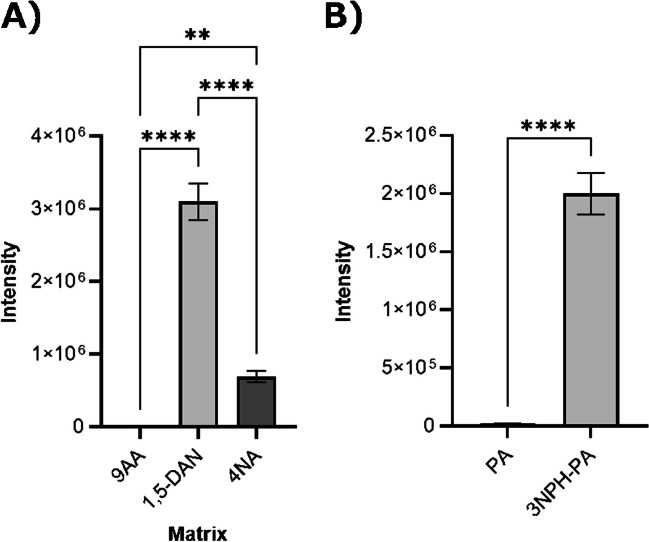
3NPH addition increased the sensitivity of pyruvate detection. **A** Comparison of the peak intensities of different matrix types, namely 9-aminoacridine, 1,5-diaminonaphthalen (1,5-DAN), and 4-nitroaniline, mixed after the 3NPH addition reaction. Peak intensities were significantly higher under conditions where 1,5-DAN was used as the matrix. **B** Comparison of peak intensities of pyruvate and 3NPH-pyruvate when 1,5-DAN was used as matrix. *n*=3, **p<0.01, ****p<0.0001

A peak corresponding to 3NPH-PA, which formed an amide bond only with the carboxyl or carbonyl group of pyruvate, was detected (Fig. S1A). The ion spectrum of this product (*m/z* of 222.05) also showed an *m/z* of 137.03. This was a characteristic of the 3NPH addition product. The peak intensity at an *m/z* of 357.09 was significantly higher (Fig. S1B). The ratio of the intensities of the peaks at *m/z* values of 222.05 and 357.09 did not change with reaction time (data not shown). The results suggest that 3NPH is added to both functional pyruvate groups, resulting in the irreversible and stable formation of 3NPH-PA. Figure 2A shows the results of the investigation of the best matrix for the detection of 3NPH-PA. As the detection intensity of MALDI-MS is firmly matrix dependent, the detection intensity of 3NPH-PA was compared when measured with 9AA, 1,5-DAN, and 4NA, which exhibit different proton affinities and ionization potentials and are commonly used for negative-ion detection. The detection sensitivity of 3NPH-PA was significantly higher when 1,5-DAN was used as the matrix compared with those when 9AA or 4NA was used as the matrix. This was thought to be because 1,5-DAN is a strong reducing agent and its ability to remove protons is superior to that of 9AA and 4NA. Based on these results, 1,5-DAN was selected for the measurement of 3NPH-PA. 1,5-DAN has amino groups at positions 1 and 5 of the naphthalene skeleton, which could compete with 3NPH. However, no pyruvate peaks were detected when 1,5-DAN was added (data not shown). A comparison of the detection intensities of pyruvate with and without the addition of 3NPH is shown in Fig. 2B. The detection intensity of 3NPH-PA was significantly higher with the addition of 3NPH than that of pyruvate without the addition of 3NPH. These results indicated that 3NPH derivatized the carbonyl and carboxyl groups.

### Optimization of derivatization conditions for MALDI-MSI

**Fig. 3:**
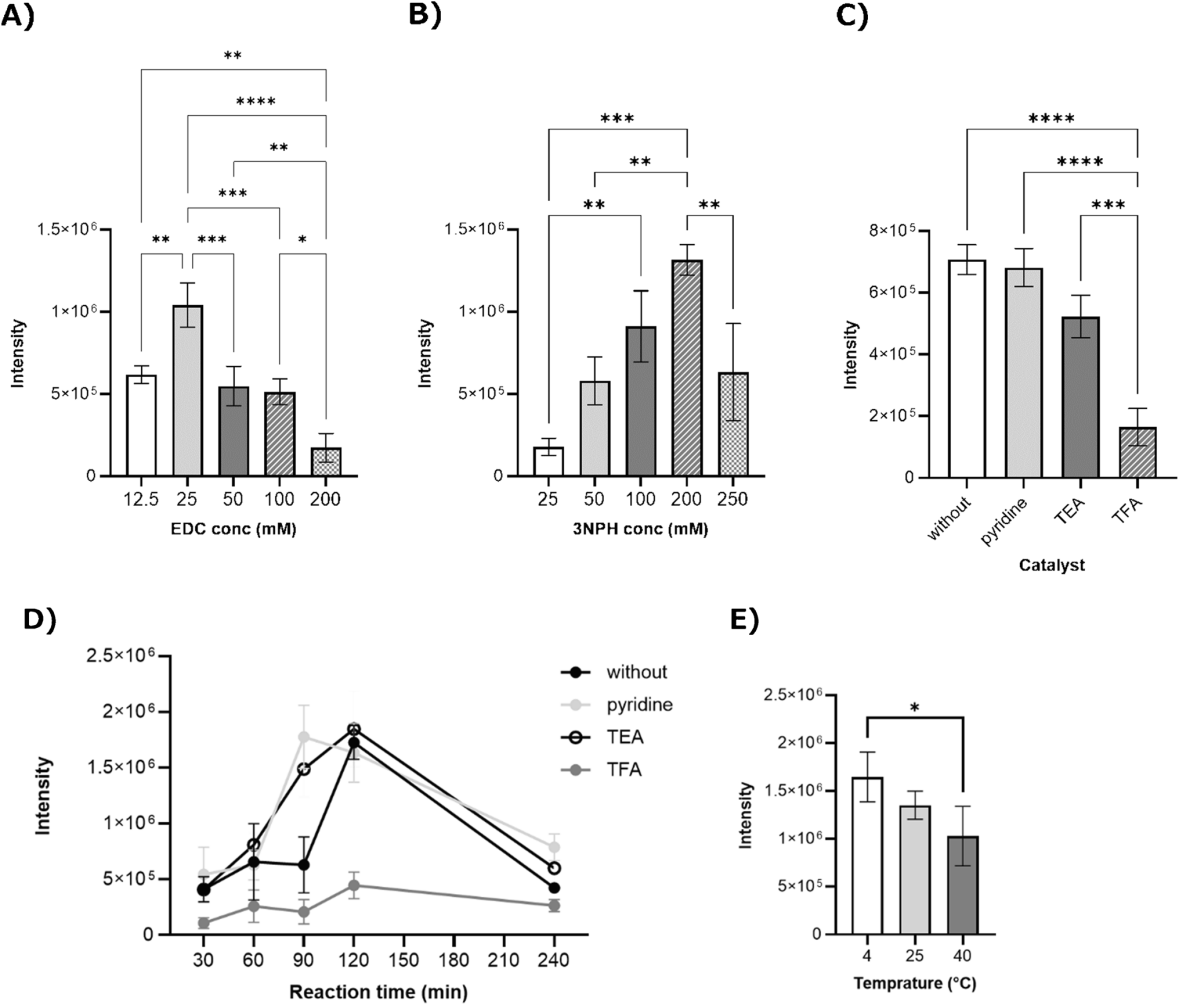
Optimization of reaction conditions. **A** EDC concentrations (12.5, 25, 50, 100, and 200 mM), **B** 3NPH concentrations (25, 50, 100, 200, and 250 mM), and **C** catalysts (pyridine, TEA, and TFA). **D** Peak intensities versus reaction time. **E** Temperature (4, r.t, and 40 °C). *n*=3, *p<0.05, **p<0.01, ***p<0.001, ****p<0.0001

The first step was to optimize the liquid-phase derivatization conditions. The peak intensities of 3NPH-PA at each EDC concentration were compared using 200 mM 3NPH and a reaction time (Fig. 3A). When the EDC concentration was 25 mM, the peak intensity of 3NPH-PA was significantly higher than those at other concentrations. The size and uniformity of the matrix crystals affect the intensity of MALDI-MSI; it was expected that the detection intensity would be lower when the amount of EDC added was excessive, as the volatility decreases with the amount of EDC added, making it more difficult for matrix crystals to form. Detection intensity was also low when the amount of EDC added was low. The peak intensities of 3NPH-PA at each 3NPH concentration were compared using 25 mM EDC (Fig. 3B). When the 3NPH concentration was 200 mM, the peak intensity of 3NPH-PA was higher than those at the other concentrations. Excess 3NPH was thought to compete and reduce derivatization efficiency. A comparison of the 3NPH-PA peak intensities for different catalysts under 25 mM EDC and 200 mM 3NPH is shown in Fig. 3C. The peak intensities of 3NPH-PA were significantly higher for pyridine and TEA than for TFA, and there was no difference in the peak intensities of 3NPH-PA for pyridine and TEA. The fact that the desorption/addition reaction by EDC was most efficient at approximately pH 5.5 and that pyruvate was on the acidic side suggested that the acidic catalyst, TFA, reduced the formation rate of NPH-PA, resulting in a lower peak intensity. Although the peak intensity of 3NPH-PA was similar to that of pyridine and TEA, even with EDC only (without), and the catalyst did not affect the peak intensity at approximately pH 5.5 [49], it was considered that it could promote the derivatization reaction and affect the reaction time. Therefore, the evolution of the 3NPH-PA peak intensity at different reaction times with the addition of each catalyst was investigated (Fig. 3D). The EDC reaction involves the formation of an amide bond via an intermediate (active ester). Under the catalyst-added conditions, the peak intensity of 3NPH-PA increased slowly until 90 or 120 min, indicating that the catalyst promoted the reaction via EDC. On the other hand, under the catalyst-free conditions, the intensity increased rapidly from 90 to 120 min, indicating a slower reaction than under the catalyst-added conditions. The results showed that the peak intensity of 3NPH-PA increased 90 min after the start of the reaction when pyridine was added and at 120 min for the other catalysts and without the catalyst. Subsequently, the detection sensitivity decreased, which was thought to be due to competition with the carbonyl groups present in the by-product of the EDC reaction, isourea. However, no significant differences in peak intensities were observed 120 min after the start of the reaction under catalytic and non-catalytic conditions, except for TFA. When the derivatization reaction had run for 120 min, the differences in the detection intensity of 3NPH-PA as a function of the reaction temperature were also investigated (Fig. 3E). The results showed a trend towards higher detection sensitivities at 4 °C. This was thought to be because the hydrolysis of EDCs in aqueous solution, which takes less time at higher temperatures, reduces the efficiency of the 3NPH addition reaction. However, no significant difference was observed between 4 °C and room temperature for the detection sensitivities. Room temperature was preferred because temperature changes before and after derivatization could alter the in vivo metabolism. These results suggested that pyridine accelerated the EDC reaction and damaged tissue sections. Moreover, there were no significant differences in the peak intensities between conditions with and without pyridine. Therefore, the method without pyridine was considered optimal. These results suggested that the optimal conditions for MALID-MSI were 25 mM EDC and 200 mM 3NPH without the addition of a catalyst. These conditions were further investigated for derivatization reactions on the ITO glass used for the MALDI-MSI measurements. Derivatized solutions with different mixing ratios of EDC and 3NPH were sprayed onto pyruvate solutions spotted onto the ITO glass, and the detection intensities of 3NPH-PA were compared (Fig. S2A). The detection intensity of 3NPH-PA was significantly higher when the EDC:3NPH ratio was 1:2 (v/v) compared to 1:1 (v/v) or 1:4 (v/v). This was thought to be due to the higher amount of EDC in 1:1 (v/v), which prevented the formation of matrix crystals. The higher amount of 3NPH in 1:4 (v/v) reduced the derivatization efficiency due to competition. Subsequently, the detection intensity of 3NPH-PA by vapor was compared (Fig. S2B) because the derivatization of dried tissue sections commonly performed in MALDI-MSI was less efficient. The results showed that the intensity of 3NPH-PA detection was significantly enhanced in a vapor-saturated environment compared to air. This suggests that the EDC desorption/incompatibility reaction is more effective in solution or under wet conditions. Because excess carboxylic acids and amines inhibited the EDC-mediated desorption/addition reaction, the amount of derivatizing reagent supplied was also investigated (Fig. S2C). The results showed that 3NPH-PA tended to be detected at higher intensities when less 3NPH was present. Based on these results, the optimum conditions for spray derivatization were determined to be saturated acetonitrile after six cycles with a rate of 5 s/cycle of the derivatizing reagent mixed with EDC:3NPH (1:2, v/v) when 200 mM 3NPH and 25 mM EDC were supplied.

### Imaging of pyruvate and lactate in a mouse testis section

**Fig. 4:**
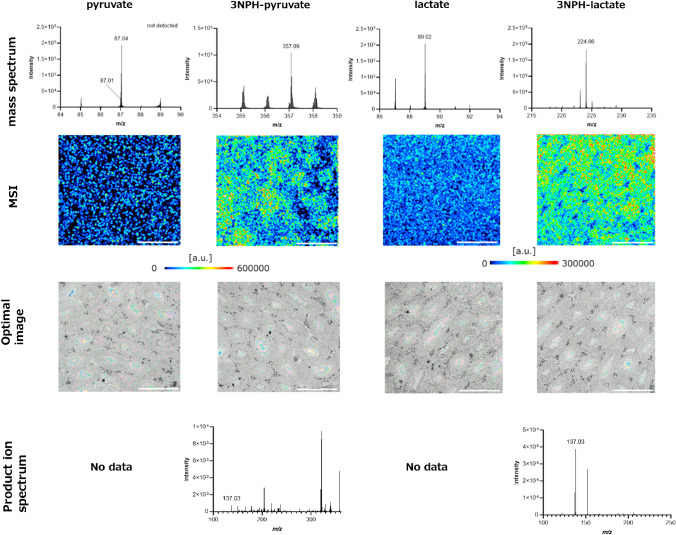
Mass spectrum, MSI and optimal image, and product ion spectrum of pyruvate and lactate in mouse testes. Scale bar: 500 µm

Pyruvate and lactate were imaged by MALDI-MSI in mouse testicular tissue sections using the derivatization method. Figure 4 shows the mass spectrum, MSI, and optimal images, product ion spectrum obtained from the mouse testicular tissue sections. The product ion spectra (*m/z* of 137.03), which are characteristic of the 3NPH-added compounds, were obtained by MSMS for 3NPH-pyruvate (*m/z* of 357.09) and 3NPH-lactate (*m/z* 224.06). This indicated that the data corresponded to compounds derivatized with 3NPH. Peaks could be detected in pyruvate and lactate without derivatization treatment, but distribution images could not be obtained for pyruvate and lactate because of a low detection intensity and significant matrix-derived peaks in the tissue, respectively. In contrast, distribution images of derivatized 3NPH-pyruvate and 3NPH-lactate in mouse testicular tissue were successfully obtained. A comparison with the results of the optical image of tissue sections after MSI measurements showed that pyruvate and lactate were mainly localized in Sertoli cells. Therefore, because pyruvate and lactate are biosynthesized in Sertoli cells [50, 51], the distribution obtained by MSI was considered reasonable. These results provide evidence that this derivatization method is effective for MALDI-MSI analysis. Visualization of the distribution of pyruvate and lactate in the testis is important because it allows quantification of the spatial extent. We have developed a quantification method using MALDI-MSI that allows objective quantification from the images obtained [52]. Observing in vivo from both a qualitative and quantitative perspective makes it possible to obtain information on where and how much of the target molecule is present in the tissue, thereby capturing changes in vivo and elucidating biological phenomena. In addition, discoveries can be made by seeing previously invisible things.

### Comparison of the peak intensities of glycolysis and TCA pathways

**Fig. 5:**
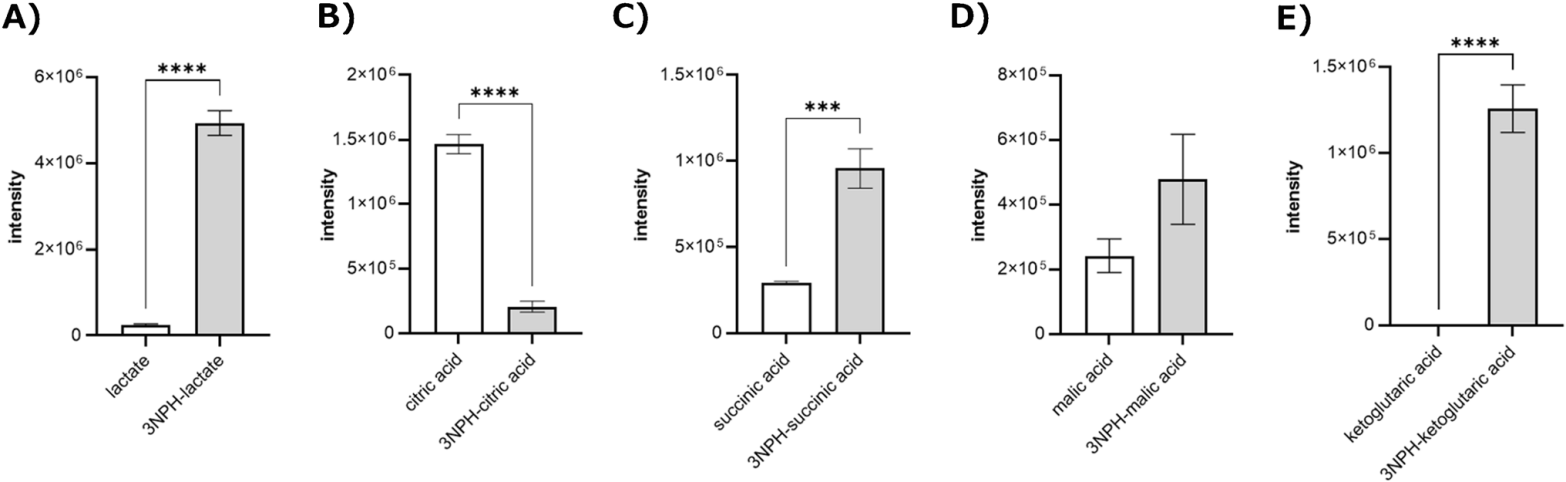
Comparison of the detection sensitivities of the main metabolites of the glycolytic and tricarboxylic acid cycles and their 3NPH adducts: **A** lactic acid, **B** citric acid, **C** succinic acid, **D** malic acid, and **E** α-ketoglutaric acid. *n*=3, ***p<0.001, ****p<0.0001

To demonstrate that this derivatization method could be used for a variety of metabolites, the peak intensities before and after derivatization were compared for lactic acid, which is a carboxyl-containing metabolite closely related to pyruvate, and metabolites of the main TCA cycle (Fig. 5). A higher sensitivity was observed for lactic acid, succinic acid, and α-ketoglutaric acid. The peak intensity of malic acid tended to increase with derivatization, whereas that of citric acid was significantly higher without derivatization. As metabolites of the TCA cycle have multiple functional groups (Fig. S3), we compared the intensities of the number of 3NPHs added (Fig. S4). As a result, the peak intensity of the product with a 3NPH was higher. For pyruvic acid, the product with two 3NPHs added was more sensitive, but this was thought to be determined by the space for 3NPHs to be added around the functional group. The detection sensitivity of the major metabolites of TCA decreased when more than one 3NPH was added, suggesting that multiple 3NPHs were unlikely to be added. Based on this result, measuring the peak after adding a carboxyl group for derivatization by 3NPH was considered appropriate. Furthermore, the lack of 3NPH-induced sensitivity to citric acid can be attributed to the structure with three relatively close carboxyl groups, which inhibited the addition of 3NPH.

## Conclusion

In this study, a novel derivatization method for pyruvate using MALDI-MSI was developed using readily available 3NPH. This derivatization method was highly sensitive to major glycolytic and TCA cycle metabolites and successfully visualized the distribution of pyruvate and lactate in mouse testes. Using this derivatization method, MALDI-MSI demonstrated that metabolites containing carboxyl groups could be visualized. Furthermore, 3NPH formed amide bonds with carbonyl and phosphate groups, in addition to carboxyl groups, suggesting the possibility of visualizing its distribution in many metabolites.

## Supplementary Information

Below is the link to the electronic supplementary material.

Supplementary file1 (PDF 351 KB)
