# Supplementary material for: Tissue derivatization for visualizing lactate and pyruvate in mouse testis tissues using matrix-assisted laser desorption/ionization-mass spectrometry imaging
Source: Anal Bioanal Chem. 2024 Oct 8;416(28):6601–10. doi: 10.1007/s00216-024-05559-4 (PMC11541321; doi:10.1007/s00216-024-05559-4)
Supplement: Supplementary file 1 — Supplementary file1 (PDF 351 KB) [file 216_2024_5559_MOESM1_ESM.pdf]

Supporting information for

"Tissue derivatization for visualizing lactate and pyruvate in mouse testis tissues using matrix-assisted laser desorption/ionization-mass spectrometry imaging."

Erika Nagano[1], Kazuki Odake[1] and Shuichi Shimma[1,2,3*]

1. Miruion inc, 7-7-20Asagi, Saito, Suita, Osaka, 5670085, Japan
2. Department of Biotechnology, Graduate School of Engineering, Osaka University, 2-1 Yamadaoka, Suita, Osaka, 5650871, Japan
3. Institute for Open and Transdisciplinary Research Initiatives, Osaka University

Figure. S1

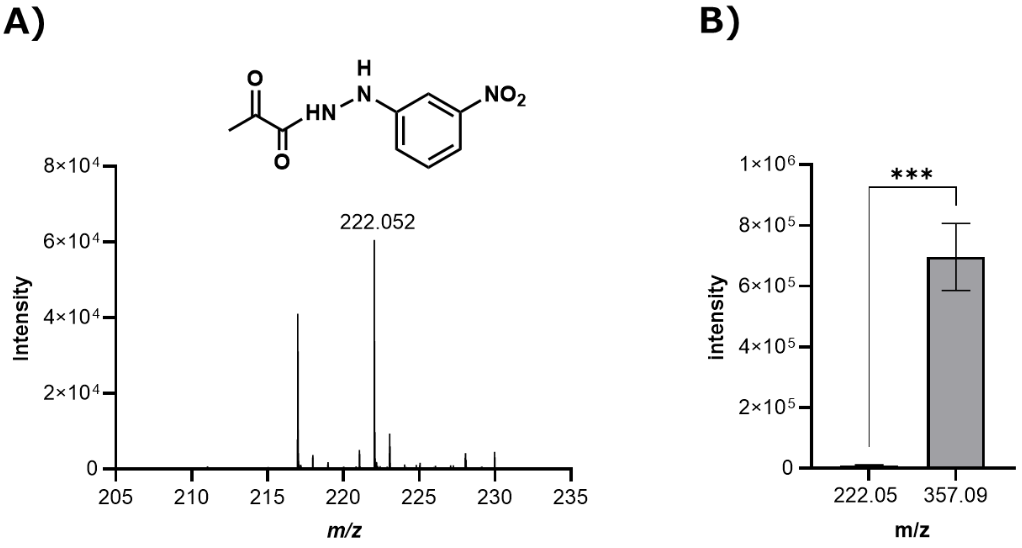

Figure. S1 A) Structural formula and mass spectrum of the derivatization product of pyruvate with 3NPH added to the carboxyl group only B) Comparison of the detection sensitivity of the product with 3NPH added to the carboxyl or carbonyl group only (*m/z* 222.05) and the product with 3NPH added to the carbonyl and carboxyl groups (*m/z* 357.09). n=3, ***p<0.001.

Figure. S2

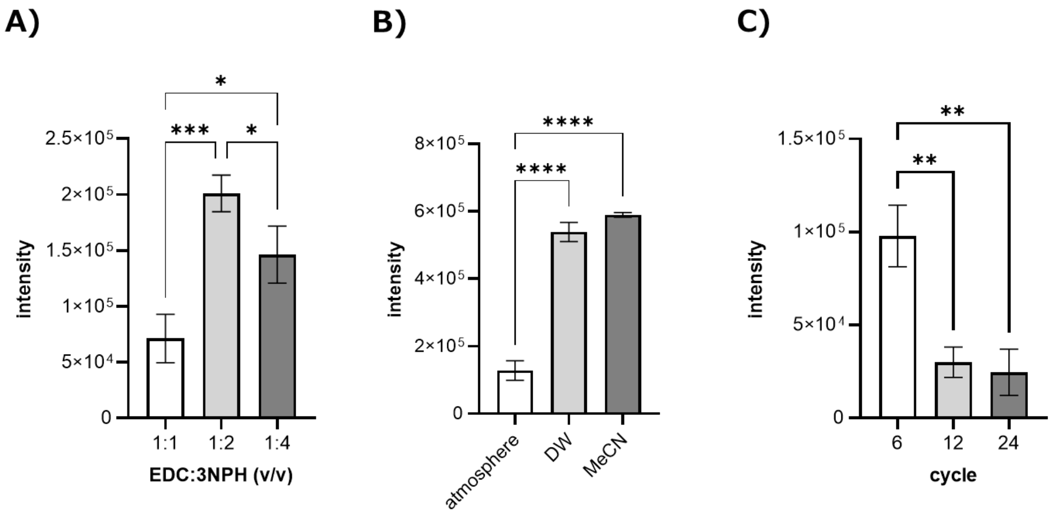

Figure. S2 Optimization study of spray method conditions using 3NPH-PA. A) Comparison of peak intensities for different mixing ratios of EDC and 3NPH solutions B) Comparison of peak intensities for different gas phase conditions of the derivatization reaction C) Comparison of peak intensities for different feed volumes of the derivatization reagent; each cycle of 5 s was used as one cycle for each number of feeds. n=3, *p<0.05, **p<0.01, ****p<0.0001.

Figure. S3

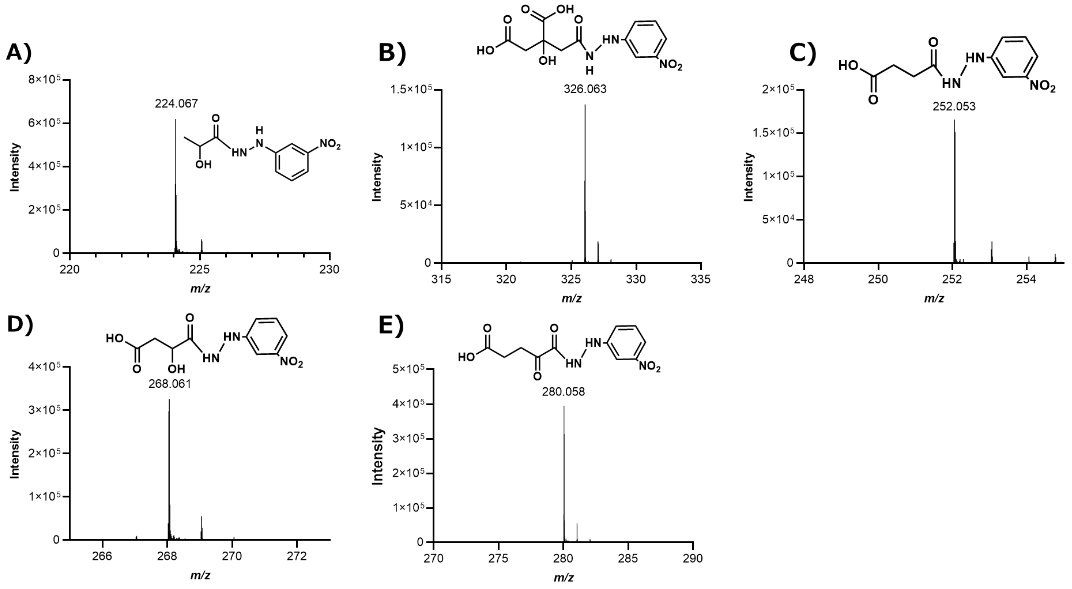

Figure. S3 Structure and mass spectra of intermediate metabolites of the lactic acid and TCA cycles. A) Lactic acid, B) Citric acid, C) Succinic acid, D) Malic acid, E) alpha-ketoglutaric acid.

Figure. S4

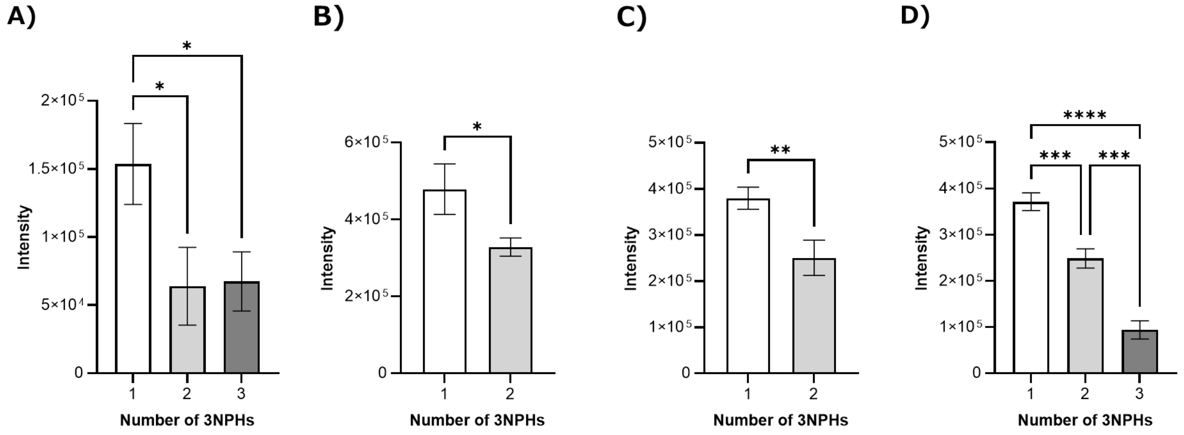

Figure. S4 Comparison of peak intensities of the number of 3NPHs added. A) Citric acid, B) Succinic acid, C) Malic acid, D) alpha-ketoglutaric acid. n=3, *p<0.05, **p<0.01, ***p<0.001.
